# The Combined Effect of Poor Perceived Indoor Environmental Quality and Psychosocial Stressors on Long-Term Sickness Absence in the Workplace: A Follow-Up Study

**DOI:** 10.3390/ijerph16244997

**Published:** 2019-12-09

**Authors:** Eerika Finell, Jouko Nätti

**Affiliations:** Faculty of Social Sciences, Tampere University, 33014 Tampere, Finland; jouko.natti@tuni.fi

**Keywords:** indoor environmental quality, social support from supervisors, experiences of injustice, discrimination, occupational health, sickness absence

## Abstract

Background: Poor perceived indoor environmental quality (IEQ) can generate conflicts and experiences of injustice in workplaces. Therefore we examined whether the combined effect of poor IEQ and self-reported psychosocial stressors (low social support from supervisors and experiences of injustice) increase the risk of employees’ long-term sickness absence (more than 10 days) in comparison to employees who report only poor perceived IEQ and no psychosocial stressors. Methods: Using negative binomial modelling, we analysed a representative sample of the working-age population in Finland (N = 16,084) from the Finnish Quality of Work Life Surveys (FQWLS) from 1997, 2003, 2008 and 2013, combined with register-based follow-up data on employees’ long-term absences covering a period of one to three years after each FQWLS was collected. Results: After background variables were included in the model, employees who reported poor IEQ and low social support had 1.18 (incidents rate ratios; 95% CI 1.05–1.33) higher rates of long-term absence than those who reported poor IEQ and high support. Similarly, employees who reported poor IEQ and experiences of injustice had 1.31(incidents rate ratios; 95% CI 1.15–1.48) higher rates of absence than those who reported poor IEQ and no injustice. Conclusions: Employees who reported poor perceived IEQ and a psychosocial stressor had higher rates of long-term sickness absence one to three years later, in comparison with those who report only poor perceived IEQ and no psychosocial stressors. These findings demonstrate the importance of taking account of psychosocial stressors as well, when resolving indoor environmental problems.

## 1. Introduction

Both poor perceived indoor environmental quality (perceived IEQ) and problems regarding the psychosocial work environment can increase sickness absences from work [1,2,3]. Poor perceived IEQ is often related to poor ventilation, mould and damp, indoor temperatures that are too low or high, outdoor pollution, chemicals in building materials and inadequate cleaning [4]. These factors can lower employees’ comfort at work, impair their cognitive performance, or increase their risk of respiratory illnesses and mucosal symptoms, weakening employees’ productivity and increasing sickness absences [5,6,7,8,9,10]. It is estimated that increasing ventilation rates to 15 litres per second per person would avoid 10 million days of short-term absence in the USA annually, leading to savings of 3.2 billion dollars [11]. In addition, reducing just 30% of the damp and mould in US office buildings would result in an annual reduction of 1.5 million absence days [11]. In Finland, which has 5.5 million inhabitants, the annual cost of short-term absence days associated with mould and damp is 403 million euros [12].

In addition to poor perceived IEQ, problems in the psychosocial work environment can also increase employees’ sickness absences. In particular, unsupportive supervision and experiences of injustice and discrimination increase this risk [2,3,4,5,6,7,8,9,10,11,12,13,14]. It is well known that social support reduces stress, whereas social conflicts and experiences of injustice increase it [15,16,17]. People who already feel themselves as threatened might be especially vulnerable to the negative effects of such stress. For example, musculoskeletal injury patients’ perceived injustice predicted their persistence of post-traumatic stress symptoms, depressive symptoms, intensity of pain and probability of returning to work [18,19,20].

Sometimes poor perceived IEQ and poor psychosocial work environment are present simultaneously. Poor perceived IEQ can generate psychosocial problems because the former can be controversial, and the inspection and reparation processes are often slow and costly. For example, people suffering from observed or suspected indoor environmental (IE) problems in the workplace report more experiences of injustice than those who do not suffer from such strains [21]. In addition, there are some evidence suggesting that conflicts in workplaces are relatively common in such settings [22]. It follows that employees can suffer from stress caused by poor perceived IEQ, and stress caused by poor psychosocial work environment (i.e., psychosocial stress) at the same time. It is possible that this combined effect may have especially adverse health effects, and it may increase the risk of sickness absence compared with situations where these stressors appear independently. Karvala and colleagues have already demonstrated that low co-operation from supervisors increases the risk of early work withdrawal among patients who suffer from diseases that are potentially related to poor IEQ in the workplace [23].

The combined effect of poor perceived IEQ, and stress caused by poor psychosocial work environment on sickness absences has not been analysed before. This exploratory study aims to analyse this issue in the context of Finland. As examples of psychosocial stressors, we focus on employees’ perception of social support offered by supervisors and their experiences of injustice related to the workplace. In addition, we focus only on long-term sickness absences. A particular emphasis on long-term absence is important, because lengthy instances of sickness can increase the risk of work disability, risk of suicide, create financial problems, increase social isolation, reduce self-esteem and decrease career opportunities [24,25,26]. Unlike many other studies that analyse the effect of IEQ on sickness absence, we rely not on self-reported absences but on register-based data.

Our main hypothesis is that employees who report both poor perceived IEQ and low social support/experiences of injustice will have higher rates of sickness absence than those who report only poor perceived IEQ and no psychosocial stressors.

## 2. Materials and Methods

### 2.1. Data and Participants

The data consists of two linked data sets. The first is the Finnish Quality of Work Life Survey (FQWLS) conducted by Statistics Finland since 1977, which reflects views about working conditions from employees in all sectors and occupations in Finland [27,28]. Our study uses the surveys collected in 1997, 2003, 2008 and 2013. The total original sample size for these four years was 22,600 wage and salary earners. From this, a sample of 16,351 individuals were interviewed face-to-face, with a response rate of 72% [28]. All the information we use in our analysis, except that related to employees’ long-term absences, is derived from FQWLS.

Our second source is the register-based follow-up data on the entire Finnish population maintained by Kela, the Finnish social insurance institution. Kela keeps records on sickness allowances paid for medically certified absences longer than 10 days. Sickness allowance can be granted for a maximum period of 300 working days per annum. Maternity leave and absences from work to care for sick children are not included in the absence register.

The two data sets were merged using the participants’ personal security numbers, a process approved by the Ethical Committee of Statistics Finland and performed by Statistics Finland (TK-53-1237-17). The final data does not include any information that compromises the anonymity of the participants, and all the ethical standards set by Statistics Finland were followed throughout our study. In the final analysis, we included only participants aged 18–62 years. The final sample size was 16,084 participants, and the missing values varied between 0% and 1.2%.

### 2.2. Measures

#### 2.2.1. Outcome Variable

We drew *long-term absence due to sickness* information from the register data maintained by Kela. This measure corresponds to the total number of days a participant was absent from work because of long-term illness (i.e., absences that exceeded 10 days) over a period of three years. We used this number as our outcome variable, limiting the follow-up to a period which started one calendar year after FQWLS was collected and ended three years later (e.g., if FQWLS was collected in 2013, the follow-up years were 2014, 2015 and 2016). These accumulated long-term absences may have been the result of one or several sickness periods exceeding 10 days, and were not necessarily consecutive. The register data does not include information about the number of absence periods.

#### 2.2.2. Predictors

*Perceived IEQ* was measured by two sets of items. The first set of eight items asked whether there were problems with the workplace indoor environment (heat; cold; draught; smoke, gases and fumes; humidity; dust; mould in the work environment; inadequate ventilation in 2003, 2008 and 2013/dry air in 1997). The second set asked about the degree to which these problems put a strain on participants at work: the five response options varied between ‘very much’ and ‘not at all’. We combined these two sets of items and recategorised the new dichotomised variables. The value *0* meant that there were no problems in the workplace indoor environment: participants considered these problems to be only a minor strain or no strain at all (i.e., good IEQ). The value *1* meant that participants reported that at least one of these problems was either ‘quite a lot’ or ‘very much’ of a strain (i.e., poor IEQ).

*Perceived social support from supervisors* was measured by 10 items (e.g., ‘my superior supports and encourages me’; ‘my superior trusts his/her employees’; ‘my superior rewards good work performance’). The five response options varied between ‘totally agree’ and ‘totally disagree’. The items were summed and divided by the number of items (scale 1–5, mean = 3.80 and standard deviation (SD) = 0.81). Then, the summed variable was recategorised into two categories, using the mean of all years (3.80) as a cut value (0 = high social support; 1 = low social support).

*Experiences of injustice* were measured by six items asking whether participants had been subjected to unequal treatment or discrimination in the workplace (0 = no, 1 = yes) in the previous five years, in various contexts (e.g., attitudes of co-workers or superiors; when receiving information; at time of hiring or appointment). These six items were summed (scale 0–6, mean = 0.64, SD = 1.15) and recategorized with the cut point of 0: the value 0 meant that there was no experience of injustice during the previous five years; the value 1 meant that participants reported at least one type of experience of injustice.

Finally, we formed two new combined variables. First, we combined perceived IEQ and social support, as follows: 1 = good IEQ and good support, 2 = good IEQ and low support, 3 = poor IEQ and good support, 4 = poor IEQ and low support. Second, we combined perceived IEQ and experiences of injustice, as follows: 1 = good IEQ and no experiences of injustice, 2 = good IEQ and experiences of injustice, 3 = poor IEQ and no experiences of injustice, 4 = poor IEQ and experiences of injustice.

#### 2.2.3. Background Variables

Demographic and work-related variables were selected on the basis of the expansive literature [29]. The demographic variables included *gender*, *age*, *marital status*, *at least one child under 18 years at home* and *level of education*. In addition, we controlled for *participants’ perceived control over job tasks*, *physical demands of job tasks*, *mental demands of job tasks* and *year of data collection*. We used as our health measure the number of *long-term absence days in the previous year* (i.e., from when the FQWLS data was collected).

### 2.3. Statistical Analyses

The relationships of the all variables with perceived IEQ, social support from supervisors and experiences of injustice were examined using cross-tabulations and analyses of variance. The main effects of perceived IEQ, social support from supervisors and experiences of injustice on long-term absence were analysed using a negative binomial model, as were their combined effects: (a) perceived IEQ and social support from supervisors; (b) perceived IEQ and experiences of injustice. In the combined variables, poor perceived IEQ and high social support/no experiences of injustice were used as reference groups because our main hypothesis was as follows: employees who report both poor perceived IEQ and psychosocial stressors will have higher rates of long-term sickness absence than those who report only poor perceived IEQ and no psychosocial stressors.

The days of long-term absence were clearly overdispersed: the variance was higher than the mean, and there was an excess of zeroes, indicating that a simple Poisson model was unsuitable for the analysis [30]. In the case of days of absence, the negative binomial model was more appropriate than Poisson, because the events of interest were not independent [31]. The results are represented as incidence rate ratios (IRR) with 95% confidence intervals (95% CI) and estimated marginal means of accumulated sickness days. We adjusted all the analyses for demographic factors (gender, age, marital status, dependent children, level of education), work-related factors (perceived control over the job, physical and mental demands) and a health-related factor (previous absence days). The survey year was also added to these models. The data were analysed by IBM SPSS Statistics for Windows, version 25.0 (IBM, Armonk, NY, USA) [32].

## 3. Results

The majority of the participants were women, were aged 30–44 years, and had a secondary education. Table 1 presents the frequencies and means of all the variables, as well as their associations with the predictors and the outcome variable.

In total, 62% of participants reported that at least one IEQ factor (e.g., humidity, inadequate ventilation, dust, mould) in the workplace caused them at least ‘quite a lot’ of strain. These reports decreased significantly over the years surveyed. The proportion was 65% in 1997 and 2003, 62% in 2008, and finally, 59% in 2013. Perceived IEQ was significantly associated with all of the background variables, except for gender, age and mental demands of the job. Participants who were neither cohabiting nor married reported significantly lower perceived IEQ (64%) than those who were cohabiting or married (61%). Similarly, those who had no children under 18 at home reported worse IEQ (63%) than those who had such children (61%). In addition, 69% of participants with primary and secondary educations reported that IEQ caused them strain, whereas only 51% of those with tertiary educations reported the same. Finally, participants who had low levels of control over their work, and those whose work was physically demanding, showed the highest incidence of reports that IEQ caused them strain at work.

The mean of long-term absences during the three-year follow-up period was 14.06 days (SD = 44.28; minimum = 0, maximum = 507). This value excludes the period prior to the long-term absence. Long-term absences were significantly associated with all the background variables, except for marital status and survey year. Women had more long-term absence days (mean = 15.29, SD = 44.72) than men (mean = 12.65, SD = 43.75). The highest long-term absences included participants who belonged to the oldest age group, were the least educated, had no children under 18 at home, had the least control over their work, and worked in physically or mentally demanding jobs.

Social support from supervisors was significantly associated with all the background variables, except for gender and whether the participant had children under 18 at home. The lowest social support was reported by participants who belonged to the 45–55 age group, were not cohabiting or married, were the least educated, had the least control over their work, and worked in physically or mentally demanding jobs. Perceived social support from supervisors improved significantly after 2003.

Finally, experiences of injustice were significantly associated with all the background variables except physical demands of the job. Participants who were female, were aged 30–44, were unmarried, had children under 18 at home, had tertiary educations, had the least control over their work, and worked in mentally demanding jobs reported the most experiences of injustice. The highest levels of experiences of injustice were reported in 2003 and 2008 (34%); the lowest was in 1997 (30%).

All the predictors were significantly associated. Participants who perceived their workplace IEQ as causing them at least ‘quite a lot’ of strain reported significantly more experiences of injustice (37%) than those who did not have such IEQ problems (26%). Similarly, those who perceived IEQ to be poor in their workplace reported receiving significantly less social support (51%) than those who did have not such a perception (36%). Finally, 64% of those who had experienced injustice also reported low social support from supervisors, whereas only 37% of those with no experiences of injustice reported low social support.

Table 2 presents the unadjusted and adjusted combined effects of perceived IEQ and social support from supervisors on long-term sickness absence, using a reference group: poor perceived IEQ and high social support (the main effects are presented in Table S1 in Supplementary Materials). In the unadjusted models, participants who reported low social support from supervisors and poor IEQ had higher rates of long-term absence days (estimated marginal mean = 17.98 days, standard error (SE) = 0.76) than those who reported poor perceived IEQ and high social support (estimated marginal mean = 14.50 days, SE = 0.63). All the mean differences between this category and other combined categories were also significant. After all the background variables were inserted into the model, the means of participants who reported both low social support and poor IEQ (estimated marginal mean = 13.61 days, SE = 0.58) still differed significantly from all the other categories. In addition, those who reported good social support and poor IEQ (estimated marginal mean = 11.52 days, SE = 0.50) differed significantly from those who reported good social support and good IEQ (estimated marginal mean = 9.23 days, SE = 0.45), but not from those who reported low social support and good IEQ (estimated marginal mean = 10.70 days, SE = 0.69). The two categories with good IEQ did not differ significantly from each other. There were no statistically significant interactions between social support and perceived IEQ in the unadjusted (Wald test χ^2^ (1) = 0.099, *p* = 0.753) or fully adjusted models (Wald test χ^2^ (1) = 0.039, *p* = 0.843). 

Finally, Table 3 presents the unadjusted and adjusted combined effects of perceived IEQ and experiences of injustice on long-term sickness absences using a reference group: poor perceived IEQ and no experiences of injustice (the main effects of these variables are presented in Table S1 in the Supplementary Materials). In the unadjusted model, participants who reported poor IEQ and experiences of injustice had higher rates of long-term absence days (estimated marginal mean = 18.38 days, SE = 0.92) than those who reported poor IEQ and no experiences of injustice (estimated marginal mean = 15.04 days, SE = 0.57). All the mean differences between the category poor IEQ and experiences of injustice and the other combined categories were also significant. After all the background variables were inserted into the model, the means of participants who reported both poor IEQ and experiences of injustice (estimated marginal mean = 14.72 days, SE = 0.73) still differed significantly from all the other categories. In addition, those who did not report experiences of injustice but reported poor IEQ (estimated marginal mean = 11.27 days, SE = 0.44) differed significantly from those who reported neither experiences of injustice nor poor IEQ (estimated marginal mean = 9.19 days, SE = 0.42). However, they did not differ from those who reported experiences of injustice and good IEQ (estimated marginal mean = 11.70 days, SE = 0.89). Those who did not report any stressors differed significantly from all the other categories. There were no statistically significant interactions between experiences of injustice and perceived IEQ in the unadjusted (Wald test χ^2^ (1) = 1.15, *p* = 0.284) or fully adjusted models (Wald test χ^2^ (1) = 0.03, *p* = 0.858).

## 4. Discussion

We found that employees who reported low social support from supervisors and poor perceived IEQ had higher rates of long-term sickness absence one to three years later, compared with those who reported only one or no stressor. Similarly, we found that employees who reported experiences of injustice and poor perceived IEQ had the highest rates of long-term sickness absence in comparison with other categories. In addition, our results show that employees who suffered either from poor perceived IEQ only, or from a psychosocial stressor only, had similar numbers of absence days. Finally, those who reported poor perceived IEQ but no psychosocial stressors had higher rates of long-term sickness absence one to three years later compared with those who did not report any stressor. These findings are in line with previous research showing that low social support from supervisors/experiences of injustice and poor IEQ are related to absences from work [1,3,10,13]. Our study contributes to this research by focusing on the combined effect of these factors, which has not been done before. In addition, we focus on long-term sickness absences, which previous research on IEQ has rarely analysed.

The important task for future research is to better understand why the combined effect of perceived IEQ and psychosocial stressors on employees’ health is greater than the effects of these environmental and psychosocial stressors taken separately. Suspected or observed IE problems in the workplace may be perceived as threats which produce stress. Social support is especially important in such contexts, because it helps people to cope [15,16,33]. However, as research on slow-moving environmental disasters has largely demonstrated, such problems are often accompanied by social conflicts between authorities and members of the community as well as between community members; they run the risk of dividing into ‘believers’ and ‘non-believers’ [34,35]. This disrupts community cohesion and increases members’ alienation. These threatening and ambivalent situations can increase depression, anxiety and post-traumatic stress [35,36,37]. Although suspected or observed IE problems are usually much less severe, these problems too may be accompanied by unsupportive supervision and experiences of injustice [21,38]. This can make it difficult to get support when it is particularly needed and it may, in turn, negatively affect the employee’s health [39,40,41].

Our results show that supervisors’ role is especially important in attempts to minimise employees’ long-term absences in the context of IE problems. Supervisors are often in a key position to provide emotional and material support to employees, and they play a crucial role in establishing organisational norms [42]. The practical implication of this study is that supervisors, occupational health practitioners and other authorities should be aware of the importance of fair and supportive treatment when they are resolving suspected or observed IE problems in the workplace. Besides the careful investigation and proper remediation of such problems [43], it is important to minimize the related negative psychosocial consequences. In addition, special attention should be given to open, respectful, and regular organizational communication [44]. IE problems raise concerns [45], and being worried may also affects people’s well-being [46].

Our study design had many advantages. We had a large and representative body of data on employees from all sectors and occupations, with a very low number of missing values. Our information on long-term sickness absences was based on nationwide register data, so we were not reliant upon self-reported absence rates, which are common in this research field. In addition, our design had a temporal component, which allowed us to test, for the first time, whether perceived IEQ and its effects when combined with psychosocial stressors were related to employees’ long-term absences one to three years later.

Given that we did not have any physical measurements, our study also had obvious limitations. However, we would argue that this limitation ought not to be considered a fundamental problem. A cumulative body of research has shown that people can evaluate IEQ relatively accurately [47,48,49]. However, psychosocial factors can also influence perceived IEQ and, therefore, questionnaires cannot be the only tool to evaluate it [50,51]. Other limitations are that we did not have measurements of building-related health symptoms and the diagnoses of those participants who had long-term absences remained unknown to us. According to the statistics provided by Kela, the commonest reasons for sickness allowance in 2017 were musculoskeletal diseases (29%), mental and behavioural disorders (21%), external causes (e.g., fractures, 15%) and respiratory diseases (6%) [52]. Finally, our model did not include information from different occupational sectors and countries other than Finland; see more information about sickness absence systems in different countries [53]. Thus, future research is needed to replicate our findings using additional tools of measurement, data from other countries and information on diagnoses and occupational sectors. 

## 5. Conclusions

Our analyses show that employees who reported both poor perceived IEQ and a psychosocial stressor had the highest rates of long-term sickness absence one to three years later in comparison with those who reported only an environmental stressor, only a psychosocial stressor, or no stressor at all. This finding suggests that environmental and psychosocial stressors should not only be treated as separate factors in analyses, and that their combined effects should also be taken into account. Environmental problems are never only ‘environmental’, but always ‘socio-environmental’: they transform the relationship between the material world and the community [54]. Additional research is needed to replicate our findings, and to test the combined effect of IEQ and psychosocial stressors on other health indicators.

## Figures and Tables

**Table 1:** Descriptive statistics of all variables and their unadjusted associations with perceived IEQ, perceived social support from supervisors, experiences of injustice, and long-term sickness absence (N = 15,898–16,084).

Variables	N	% or Mean (SD)	X^2^/F-Test Predictor: Perceived IEQ	X^2^/F-Test Predictor: Social Support	X^2^/F-Test Predictor: Experiences of Injustice	F-Test Outcome Variable: Long-Term Absence
Gender (female)	8577	53	0.30	1.17	215.25 ***	14.27 ***
Age (years) 18–29	2885	18	0.40	68.33 ***	58.20 ***	77.34 ***
30–44	6041	38				
45–54	4567	28				
55–62	2591	16				
Marital status (cohabiting/married)	11676	73	7.89 **	7.85 **	4.83 *	0.43
Children under 18 (yes)	6521	41	5.86 *	0.11	12.62 ***	31.21 ***
Level of education Primary	2507	16	540.35 ***	22.19 ***	88.34 ***	70.65 ***
Secondary	7437	46				
Tertiary	6140	38				
Year 1997	2954	18	49.03 ***	41.17 ***	17.57 **	1.12
2003	4070	25				
2008	4332	27				
2013	4728	29				
Perceived control over job						
Low	2483	15	185.16 ***	424.71 ***	148.47 ***	18.38 ***
Medium	8155	51				
High	5441	34				
Physical demands of job tasks (demanding)	5512	34	1127.18 ***	83.26 ***	2.25	149.61 ***
Mental demands of job tasks (demanding)	8108	50	1.95	98.71 ***	367.45 ***	11.48 **
Baseline absenteeism (days)	16084	3.09 (15.26) ^a^	25.83 ^b^ ***	20.13 ^b^ ***	21.44 ^b^ ***	7.15 ***
Perceived IEQ (poor)	9984	62	-	336.60 ***	200.99 ***	66.32 ***
Social support from supervisor (low)	7250	45	336.60 ***	-	1005.14 ***	27.34 ***
Experiences of injustice (yes)	5270	33	200.99 ***	1005.14 ***	-	29.40 ***
Long-term absence (days)	16084	14.06 (44.28) ^a^	66.32 ^b^ ***	27.34 ^b^ ***	29.40 ^b^ ***	-

*** < 0.001, ** < 0.01, * < 0.05.^a^ Mean and standard deviation. ^b^ F-test.

**Table 2:** Unadjusted and adjusted negative binomial models of combined effect of perceived IEQ and social support from supervisors predicting long-term sickness absence (N = 15,884–15,896).

**Unadjusted Model** **Support from Supervisor**	**N**	**%**	**IRR ^a^**	**95% CI**	**Estimated Marginal Means**	**Pairwise Comparisons**
1. Good IEQ + high support	3827	24	0.67 ***	0.59–0.76	9.73	1 < 2 *; 1 < 3 ***; 1 < 4 ***
2. Good IEQ + low support	2182	14	0.81 **	0.69–0.94	11.68	2 > 1 *; 2 < 3 **; 2 < 4 ***
3. Poor IEQ + high support	4819	30	1		14.50	3 > 1 ***; 3 > 2 **; 3 < 4 ***
4. Poor IEQ + low support	5068	32	1.24 ***	1.10–1.40	17.98	4 > 1 ***; 4 > 2 ***; 4 > 3 ***
**Adjusted Model (Demographics) ^1^** **Perceived IEQ and Social Support from Supervisor**	**N**	**%**	**IRR**	**95% CI**	**Estimated Marginal Means**	**Pairwise Comparisons**
1. Good IEQ + high support	3825	24	0.69 ***	0.60–0.78	8.80	1 < 2 *; 1 < 3 ***; 1 < 4 ***
2. Good IEQ + low support	2182	14	0.84 *	0.72–0.98	10.85	2 > 1 *; 2 < 3 *; 2 < 4 ***
3. Poor IEQ + high support	4818	30	1		12.85	3 > 1 ***; 3 > 2 *; 3 < 4 **
4. Poor IEQ + low support	5068	32	1.21 **	1.07–1.36	15.50	4 > 1 ***; 4 >2 ***; 4 > 3 **
**Adjusted Model (Demographics and Work Characteristics) ^2^** **Perceived IEQ and Social Support from Supervisor**	**N**	**%**	**IRR**	**95% CI**	**Estimated Marginal Means**	**Pairwise Comparisons**
1. Good IEQ + high support	3823	24	0.77 ***	0.68–0.88	9.35	1 < 3 ***; 1 < 4 ***
2. Good IEQ + low support	2177	14	0.91	0.78–1.06	10.99	2 < 4 ***
3. Poor IEQ + high support	4817	30	1		12.13	3 > 1 ***; 3 < 4 **
4. Poor IEQ + low support	5067	32	1.18 **	1.05–1.33	14.29	4 > 1 ***; 4 > 2 ***; 4 > 3 **
**Fully Adjusted Model (Demographics, Work Characteristics and Baseline Absenteeism) ^3^** **Perceived IEQ and Social Support from Supervisor**	**N**	**%**	**IRR**	**95% CI**	**Estimated Marginal Means**	**Pairwise Comparisons**
1. Good IEQ + high support	3823	24	0.80 **	0.71–0.91	9.23	1 < 3 **; 1 < 4 ***
2. Good IEQ + low support	2177	14	0.93	0.80–1.08	10.70	2 < 4 **
3. Poor IEQ + high support	4817	30	1		11.52	3 > 1 **; 3 < 4 **
4. Poor IEQ + low support	5067	32	1.18 **	1.05–1.33	13.61	4 > 1 ***; 4 > 2 **; 4 > 3 **

*** < 0.001, ** < 0.01, * < 0.05. ^a^ IRR = incidence rate ratios. ^1^ Adjusted model controlled for: gender, age, marital status, children under 18, education, year data was collected. ^2^ Adjusted model controlled for: gender, age, marital status, children under 18, education, year data was collected, perceived control over job tasks, physical and mental demands of job tasks. ^3^ Fully adjusted model controlled for: gender, age, marital status, children under 18, education, year data was collected, perceived control over job tasks, physical and mental demands of job tasks, baseline absenteeism.

**Table 3:** Unadjusted and adjusted negative binomial models of combined effect of perceived IEQ and experiences of injustice on long-term sickness absence (N = 16,066–16,078).

**Unadjusted Model** **Perceived IEQ and Experiences of Injustice**	**N**	**%**	**IRR ^a^**	**95% CI**	**Estimated Marginal Means**	**Pairwise Comparisons**
1. Good IEQ + no experiences of injustice	4509	28	0.63 ***	0.56–0.71	9.51	1 < 2 **; 1 < 3 ***; 1 < 4 ***
2. Good IEQ + experiences of injustice	1589	10	0.87	0.73–1.02	13.04	2 > 1 **; 2 < 4 ***
3. Poor IEQ + no experiences of injustice	6300	39	1		15.04	3 > 1 ***; 3 < 4 **
4. Poor IEQ + experiences of injustice	3680	23	1.22 **	1.08–1.38	18.38	4 > 1 ***; 4 > 2 ***; 4 > 3 **
**Adjusted Model (Demographics) ^1^** **Perceived IEQ and Experiences of Injustice**	**N**	**N**	**IRR**	**95% CI**	**Estimated Marginal Means**	**Pairwise Comparisons**
1. Good IEQ + no experiences of injustice	4508	28	0.68 ***	0.61– 0.77	8.58	1 < 2 ***; 1 < 3 ***; 1 < 4 ***
2. Good IEQ + experiences of injustice	1588	10	1.01	0.85–1.20	12.68	2 > 1 ***; 2 < 4 **
3. Poor IEQ + no experiences of injustice	6299	39	1		12.53	3 > 1 ***; 3 < 4 ***
4. Poor IEQ + experiences of injustice	3680	23	1.35 ***	1.19–1.53	16.93	4 > 1 ***; 4 > 2 **; 4 > 3 ***
**Adjusted Model (Demographics and Work Characteristics) ^2^** **Perceived IEQ and Experiences of Injustice**	**N**	**N**	**IRR**	**95% CI**	**Estimated Marginal Means**	**Pairwise Comparisons**
1. Good IEQ + no experiences of injustice	4502	28	0.79 ***	0.70–0.89	9.24	1 < 2 **; 1 < 3 ***; 1 < 4 ***
2. Good IEQ + experiences of injustice	1587	10	1.06	0.90–1.26	12.41	2 > 1 **; 2 < 4 **
3. Poor IEQ + no experiences of injustice	6298	39	1		11.68	3 > 1 ***; 3 < 4 ***
4. Poor IEQ + experiences of injustice	3679	23	1.35 ***	1.19–1.53	15.74	4 > 1 ***; 4 > 2 **; 4 > 3 ***
**Fully Adjusted Model (Demographics, Work Characteristics and Baseline Absenteeism) ^3^** **Perceived IEQ and Experiences of Injustice**	**N**	**N**	**IRR**	**95% CI**	**Estimated Marginal Means**	**Pairwise Comparisons**
1. Good IEQ + no experiences of injustice	4502	28	0.82 **	0.72–0.92	9.19	1 < 2 *; 1 < 3 **; 1 < 4 ***
2. Good IEQ + experiences of injustice	1587	10	1.04	0.88–1.23	11.70	2 > 1 *; 2 < 4 **
3. Poor IEQ + no experiences of injustice	6298	39	1		11.27	3 > 1 **; 3 < 4 ***
4. Poor IEQ + experiences of injustice	3679	23	1.31 ***	1.15–1.48	14.72	4 > 1 ***; 4 > 2 **; 4 > 3 ***

*** < 0.0010, ** < 0.01, * < 0.05. ^a^ IRR = incidence rate ratios. ^1^ Adjusted model controlled for: gender, age, marital status, children under 18, education, year data was collected. ^2^ Adjusted model controlled for: gender, age, marital status, children under 18, education, year data was collected, perceived control over job tasks, physical and mental demands of job tasks. ^3^ Fully adjusted model controlled for: gender, age, marital status, children under 18, education, year data was collected, perceived control over job tasks, physical and mental demands of job tasks, baseline absenteeism.

## Supplementary Materials

The following are available online at https://www.mdpi.com/1660-4601/16/24/4997/s1, Table S1: Unadjusted and adjusted negative binomial models of perceived IEQ, social support from supervisors and experiences of injustice predicting long-term sickness absence (N = 15,886–16,082).
