# Supplementary material for: The Combined Effect of Poor Perceived Indoor Environmental Quality and Psychosocial Stressors on Long-Term Sickness Absence in the Workplace: A Follow-Up Study
Source: Int J Environ Res Public Health. 2019 Dec 9;16(24):4997. doi: 10.3390/ijerph16244997 (PMC6950637; doi:10.3390/ijerph16244997)
Supplement: Supplementary file 1 [file ijerph-16-04997-s001.pdf]

International Journal of *Environmental Research and Public Health*

MDPI

Article

The Combined Effect of Poor Perceived Indoor Environmental Quality and Psychosocial Stressors on Long-Term Sickness Absence in the Workplace: A Follow-Up Study

Eerika Finell [1,*] and Jouko Nätti [2]

[1] Department, School, Tampere University, 33400 Tampere, Finland
[2] Department, School, Tampere University, 33400 Tampere, Finland; jouko.natti@tuni.fi
* Correspondence: eerika.finell@tuni.fi; Tel.: +358-50-318-7646

Received: 05 November 2019; Accepted: 05 December 2019; Published: date

Supplementary materials

Table S1. Unadjusted and adjusted negative binomial models of perceived IEQ, social support from supervisors and experiences of injustice predicting long-term sickness absence (N=15,886–16,082).

Predictor: perceived IEQ					
**Unadjusted model**		N	IRR [a]	95% CI	Wald test
Perceived IEQ					
	Good IEQ	6098	1		
	Poor IEQ	9984	1.56 ***	1.42–1.72	81.62
**Adjusted model (demographics)** [1]		N	IRR	95% CI	Wald test
Perceived IEQ					
	Good IEQ	6096	1		
	Poor IEQ	9983	1.49 ***	1.35–1.64	64.57
**Adjusted model (demographics and work characteristics)** [2]		N	IRR	95% CI	Wald test
Perceived IEQ					
	Good IEQ	6089	1		
	Poor IEQ	9979	1.32 ***	1.20–1.46	29.92
**Fully adjusted model (demographics, work characteristics and baseline absenteeism)** [3]		N	IRR	95% CI	Wald test

Perceived IEQ					
	Good IEQ	6089	1		
	Poor IEQ	9979	1.28 ***	1.16–1.41	24.08
**Predictor: social support from supervisor**					
**Unadjusted model**		N	IRR	95% CI	Wald test
Social support from supervisor					
	High support	8648	1		
	Low support	7250	1.30 ***	1.18–1.43	29.07
**Adjusted model (demographics)** [1]		N	IRR	95% CI	Wald test
Social support from supervisor					
	High support	8645	1		
	Low support	7250	1.28 ***	1.16–1.40	25.99
**Adjusted model (demographics and work characteristics)** [2]		N	IRR	95% CI	Wald test
Social support from supervisor					
	High support	8642	1		
	Low support	7244	1.22 ***	1.11–1.34	16.24
**Fully adjusted model (demographics, work characteristics and baseline absenteeism)** [3]		N	IRR	95% CI	Wald test
Social support from supervisor					
	High support	8642			
	Low support	7244	1.21 ***	1.10–1.33	15.33
**Predictor: experiences of injustice**					
**Unadjusted model**		N	IRR	95% CI	Wald test
Experiences of injustice					
	No	10810	1		
	Yes	5270	1.32 ***	1.19–1.46	29.05
**Adjusted model (demographics)** [1]		N	IRR	95% CI	Wald test
Experiences of injustice					
	No	10808	1		
	Yes	5269	1.46 ***	1.32–1.61	52.81
**Adjusted model (demographics and work characteristics)** [2]		N	IRR	95% CI	Wald test
Experiences of injustice					

International Journal of *Environmental Research and Public Health*

MDPI

	No	10801	1		
	Yes	5267	1.39 ***	1.26–1.54	40.17
**Fully adjusted model (demographics, work characteristics and baseline absenteeism)** [3]		N	IRR	95% CI	Wald test
Experiences of injustice					
	No	10801			
	Yes	5267	1.33 ***	1.20–1.48	30.78

*** < .001, ** < .01, * < .05. [a] IRR = incidence rate ratios. [1] Adjusted model controlled for: gender, age, marital status, children under 18, education, year data was collected. [2] Adjusted model controlled for: gender, age, marital status, children under 18, education, year data was collected, perceived control over job tasks, physical and mental demands of job tasks. [3] Fully adjusted model controlled for: gender, age, marital status, children under 18, education, year data was collected, perceived control over job tasks, physical and mental demands of job tasks, baseline absenteeism.

© 2019 by the authors. Submitted for possible open access publication under the terms and conditions of the Creative Commons Attribution (CC BY) license (http://creativecommons.org/licenses/by/4.0/).
